# Diagnosis of Sleep Apnoea Using a Mandibular Monitor and Machine Learning Analysis: One-Night Agreement Compared to in-Home Polysomnography

**DOI:** 10.3389/fnins.2022.726880

**Published:** 2022-03-15

**Authors:** Julia L. Kelly, Raoua Ben Messaoud, Marie Joyeux-Faure, Robin Terrail, Renaud Tamisier, Jean-Benoît Martinot, Nhat-Nam Le-Dong, Mary J. Morrell, Jean-Louis Pépin

**Affiliations:** ^1^National Heart and Lung Institute, Imperial College London, Royal Brompton Hospital, London, United Kingdom; ^2^HP2 Laboratory, Inserm U1300, Grenoble Alpes University, Grenoble, France; ^3^EFCR Laboratory, Thorax and Vessels division, Grenoble Alpes University Hospital, Grenoble, France; ^4^Sleep Laboratory, CHU Université catholique de Louvain (UCL) Namur Site Sainte-Elisabeth, Namur, Belgium; ^5^Institute of Experimental and Clinical Research, UCL Bruxelles Woluwe, Brussels, Belgium; ^6^Sunrise, Namur, Belgium

**Keywords:** sleep apnoea, polysomnography, mandibular monitor, in-home diagnosis, one-night agreement, performance, automated machine learning analysis

## Abstract

**Background:**

The capacity to diagnose obstructive sleep apnoea (OSA) must be expanded to meet an estimated disease burden of nearly one billion people worldwide. Validated alternatives to the gold standard polysomnography (PSG) will improve access to testing and treatment. This study aimed to evaluate the diagnosis of OSA, using measurements of mandibular movement (MM) combined with automated machine learning analysis, compared to in-home PSG.

**Methods:**

40 suspected OSA patients underwent single overnight in-home sleep testing with PSG (Nox A1, ResMed, Australia) and simultaneous MM monitoring (Sunrise, Sunrise SA, Belgium). PSG recordings were manually analysed by two expert sleep centres (Grenoble and London); MM analysis was automated. The Obstructive Respiratory Disturbance Index calculated from the MM monitoring (MM-ORDI) was compared to the PSG (PSG-ORDI) using intraclass correlation coefficient and Bland-Altman analysis. Receiver operating characteristic curves (ROC) were constructed to optimise the diagnostic performance of the MM monitor at different PSG-ORDI thresholds (5, 15, and 30 events/hour).

**Results:**

31 patients were included in the analysis (58% men; mean (SD) age: 48 (15) years; BMI: 30.4 (7.6) kg/m^2^). Good agreement was observed between MM-ORDI and PSG-ORDI (median bias 0.00; 95% CI −23.25 to + 9.73 events/hour). However, for 15 patients with no or mild OSA, MM monitoring overestimated disease severity (PSG-ORDI < 5: MM-ORDI mean overestimation + 5.58 (95% CI + 2.03 to + 7.46) events/hour; PSG-ORDI > 5–15: MM-ORDI overestimation + 3.70 (95% CI −0.53 to + 18.32) events/hour). In 16 patients with moderate-severe OSA (*n* = 9 with PSG-ORDI 15–30 events/h and *n* = 7 with a PSG-ORD > 30 events/h), there was an underestimation (PSG-ORDI > 15: MM-ORDI underestimation −8.70 (95% CI −28.46 to + 4.01) events/hour). ROC optimal cut-off values for PSG-ORDI thresholds of 5, 15, 30 events/hour were: 9.53, 12.65 and 24.81 events/hour, respectively. These cut-off values yielded a sensitivity of 88, 100 and 79%, and a specificity of 100, 75, 96%. The positive predictive values were: 100, 80, 95% and the negative predictive values 89, 100, 82%, respectively.

**Conclusion:**

The diagnosis of OSA, using MM with machine learning analysis, is comparable to manually scored in-home PSG. Therefore, this novel monitor could be a convenient diagnostic tool that can easily be used in the patients’ own home.

**Clinical Trial Registration:**

https://clinicaltrials.gov, identifier NCT04262557

## Introduction

Obstructive sleep apnoea (OSA) is a major burden worldwide, affecting nearly one billion people (Benjafield et al., 2019; Grote, 2019; Lyons et al., 2020). Alongside symptoms of sleepiness, and impaired memory and mood, untreated OSA is associated with a range of cardiovascular and metabolic morbidities and increased mortality (Knauert et al., 2015; Levy et al., 2015; Reutrakul and Mokhlesi, 2017; Linz et al., 2018). Moreover, the prevalence is set to rise with ageing populations and a global obesity pandemic. Additionally, recent data supporting treatment of mild OSA has created further burden, with over half of patients with OSA experiencing a mild form of the disease (Benjafield et al., 2019; Wimms et al., 2020). Finally, an acute need has arisen to re-evaluate OSA diagnosis and treatment, due to the COVID-19 pandemic, which has reduced resources and increased waiting lists (Patel and Donovan, 2020; Schiza et al., 2021).

Attempts to expand diagnostic capacity in the face of increasing demand have utilised technological advances. In particular, portable monitors have focused on minimally invasive measurements and automated analysis, for ease of use by both patients and staff (Collop et al., 2007). Additionally, the COVID-19 pandemic has resulted in the need for disposable diagnostic monitors that can be used safely in the patients’ home, to facilitate remote healthcare pathways (Grote et al., 2020). However, despite the obvious need for new diagnostic tools, monitors must be evaluated for reliability, since issues typically occur in the classification of breathing events as central or obstructive, plus the overall event count, in the absence of sleep monitoring (Randerath et al., 2018).

Mandibular movements (MM) have been established as a surrogate bio-signal for the detection of breathing effort during sleep (Martinot et al., 2015, 2017b,^2020^). Analysis of the MM signal has enabled the identification of specific breathing patterns associated with sleep-disordered breathing (Senny et al., 2008; Maury et al., 2013, 2014; Martinot et al., 2017a). MM analysis has also been shown to differentiate between sleep and wake states, allowing for the identification of total sleep time, which may be of value in the calculation of sleep-disordered breathing indices (Senny et al., 2009, 2012; Maury et al., 2014). In a recent study, machine learning was used to homogenise the quality of the scoring of respiratory events, linked with cloud-based data transfer; this automated analysis was equivalent to that of in-laboratory PSG (Pépin et al., 2020).

The aim of the current study was to evaluate the use a novel monitor (Sunrise, Sunrise SA, Belgium) using MM for the diagnosis of OSA in real world conditions. MM and PSG data were recorded simultaneously in the patients’ home. MM was analysed automatically and compared to PSG analysed manually by experts at two clinical centres. We hypothesised that the Obstructive Respiratory Disturbance Index (ORDI)(Nordigarden et al., 2011) calculated using MM with machine learning analysis would not be significantly different to the ORDI obtained using manually scored PSG.

## Materials and Methods

### Study Design

A prospective, diagnostic, open study in 40 adult patients referred with a suspicion of OSA to a single centre (Grenoble Alpes University Hospital) was conducted. The study was approved by an independent Ethics Committee (Comité de Protection des Personnes, Sud-Ouest et Outremer III, Bordeaux, France, ID-RCB: 2019-A02965-52) and registered on Clinicaltrials.gov (NCT04262557). All 40 patients were recruited from the Grenoble centre and signed written informed consent. The study was conducted in accordance with Good Clinical Practice, and all applicable laws and regulations. This study followed the Standards for Reporting of Diagnostic Accuracy (STARD) reporting guideline.

Forty consecutive adult patients undertaking a diagnostic home sleep study for suspicion of OSA were invited to participate. Participants had to be able to use portable devices and smartphones. All 40 participants underwent an overnight PSG (the reference method) with simultaneous MM recordings using the Sunrise system (Sunrise SA, Belgium). Two visits were scheduled; the first to verify the eligibility of the patient and to collect baseline data. The second visit was at end of the study, with the patient and clinician, for sharing of the final diagnostic report.

### Overnight Sleep Study and Scoring of Polysomnography

In-home PSG was recorded with a portable acquisition system (Nox A1, ResMed, Saint-Priest Cedex, France). Measurements used to determine sleep were electro-oculogram, electroencephalogram, electromyogram, and electrocardiogram. Oxygen saturation was also monitored by a digital oximeter displaying pulse waveform (Nonin, Nonin Medical, United States). Airflow was measured using nasal pressure associated with the sum of oral and nasal thermistor signals. Respiratory effort was monitored with abdominal and thoracic bands.

Polysomnography recordings were initially scored by experts from the recruiting centre (Grenoble Alpes University Hospital, France). PSG were anonymized, converted in European data format (EDF) and sent *via* a secured platform for blinded scoring to the second reference centre (Imperial College London, United Kingdom). Scoring was performed according to the recommended criteria established by the American Academy of Sleep Medicine (AASM) Manual for the Scoring of Sleep and Associated (Berry et al., 2012). Apnoeas were defined as a complete cessation of airflow ≥ 10 s and classified as obstructive, central, or mixed according to the presence or the absence of respiratory effort. Hypopneas were scored using the AASM-recommended hypopnoea definition, requiring at least a 30% decrement in airflow lasting 10 s or longer and associated with a decrease of at least 3% in oxygen saturation as measured by pulse oximetry, or an arousal (Berry et al., 2012, 2017). ORDI was defined as the total number of obstructive respiratory disturbances accompanied by respiratory effort divided by the total sleep time (TST) (PSG-ORDI). PSG recordings were analysed blinded to the MM data and the two centres scored the PSG recordings independently.

Obstructive sleep apnoea diagnosis was established according to the third edition of the International Classification of Sleep Disorders (ICSD-3) (Sateia, 2014). Apnoea-Hypopnoea Index (AHI) thresholds of 5, 15 and 30 events/hour were used to define OSA severity levels of mild, moderate, and severe, respectively.

### Mandibular Movement Recordings and Description of the Sunrise Monitoring System

The Sunrise monitoring system is a certified medical device used for the diagnosis of sleep apnoea using MM analysis (Sunrise SA, Namur, Belgium). The MMs were monitored using inertial measurement units and data was transferred *via* Bluetooth Low Energy to a smartphone application. For more information on MM analysis see Appendix.

Participants first downloaded the application and then performed a device association, before attaching the monitor to their chin, in the mentolabial sulcus. The recorded MM data were automatically transferred from the smartphone to a cloud-based infrastructure at the end of the night. These data were then analysed using a dedicated machine learning algorithm. The algorithm identified obstructive and mixed apnoeas and hypopnoeas, plus respiratory effort–related arousals, through stereotypical MM patterns. Respiratory disturbances were identified by periods of respiratory effort ended by an arousal or an awakening. A full description of the Sunrise System and algorithm have been previously reported (Pépin et al., 2020). The MM-ORDI was defined as the total number of obstructive respiratory disturbances accompanied by respiratory effort divided by the TST, estimated from the Sunrise analytics.

### Statistical Analysis

Data analysis was conducted using scientific computing packages (numpy, scipy) in the Python programming language.

Firstly, we evaluated the agreement between the MM-ORDI and the PSG-ORDI. For this, we compared the MM-ORDI with the PSG-ORDI calculated by scorers in Grenoble and in London and we also calculated the combined PSG-ORDI by averaging the ORDI scores from the two centres. Then, we used Pearson’s linear correlation matrix and regression plots to evaluate the linear relationship between MM-ORDI and PSG-ORDI. Next, we calculated ORDI Intraclass Correlation Coefficients (ICC) using a 2-way fixed model for single measures (ICC, 3,2) to evaluate the agreement between MM-ORDI and PSG-ORDI. Additionally, we used a complete and groupwise Bland-Altman plot to estimate the 95% limits of agreement and the systematic bias of MM-derived indices compared with their PSG counterparts.

Secondly, we evaluated the diagnostic performance of MM-ORDI for OSA based on receiver operating characteristic (ROC) curves. We performed an area under the curve (AUC), and a *post hoc* analysis to optimise the cut-off points of MM-ORDI for diagnostic decisions, compared with the criterion-standard cut-off values of obstructive PSG-ORDI recommended in ICSD-3 (5 events/hour and 15 events/hour). The optimal MM cut-offs were assessed at the highest value of the Youden index (sensitivity plus specificity minus 1). Finally, we calculated the metrics of clinical utility and accuracy for the optimal detection thresholds and the post-test probability for each cut-off point recommended by the Portable Monitoring Task Force of the AASM (Collop et al., 2007).

Statistical inference was based on null-hypothesis testing at significance threshold of *p* < 0.05.

## Results

### Participants

Forty participants were recruited to the study and data from 31 participants were included in the analysis. Two participants withdrew, and there were three technical failures of PSG (poor quality signals) and four technical failures of the Sunrise device (Bluetooth connection was lost for three patients and for one patient the Sunrise sensor became disconnected). Participants were 58% men, with a mean (SD) age of 48 (15) years and body mass index (BMI) of 30.4 (7.6) kg/m^2^.

### Evaluation of the Agreement Between Mandibular Movement Monitoring System and in-Home Polysomnography for Measuring Respiratory Disturbances

**FIGURE 1:**
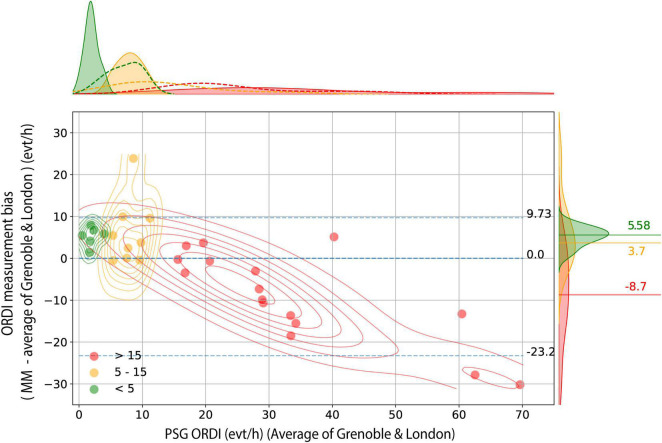
Bland-Altman analysis for MM-ORDI versus average PSG-ORDI. Bland-Altman plot shows the disagreement between average PSG-ORDI and MM-ORDI (y axis) as a function of the average PSG ORDI (x axis), with individual cases stratified into three clinical groups. Bidimensional kernel density estimation plots are superimposed to show the joint distribution of measurement bias within each subgroup. The blue horizontal lines indicate the median, lower and upper bound (5th and 95th centiles) of the measurement bias in the whole sample. The distribution of the disagreement between the two methods, stratified by group, is shown on the right, with three horizontal lines indicating the median bias within each group. MM: mandibular movement; ORDI: obstructive respiratory disturbance index; PSG: polysomnography.

The median value of PSG-ORDI, determined by averaging the ORDI scores from the two centres, was 15.45 (IQR: 1.75 to 61.38) events/hour. The median of MM-ORDI was 16.80 (IQR: 3.50 to 42.50) events/hour. Overall, there was a good agreement between MM-ORDI and PSG-ORDI with a median bias of 0.00 (95% CI −23.25 to + 9.73) events/hour (Figure 1). However, there was systematic bias across the disease severity spectrum. In patients with no OSA (<5 events/hour, *n* = 6) and mild OSA, (5–15 events/hour, *n* = 9), MM-ORDI over-estimated by a random and normally distributed bias, with medians of + 5.58 (95% CI: + 2.03 to + 7.46) and + 3.70 (95% CI −0.53 to + 18.32) events/hour, respectively. In patients with moderate-severe OSA (ORDI score > 15, *n* = 16) MM-ORDI underestimated by −8.70 (95% CI −28.46 to + 4.01) events/hour.

### Evaluation of the Agreement Between Two Expert Centres for Measuring Obstructive Respiratory Disturbance Index From in-Home Polysomnography

**FIGURE 2:**
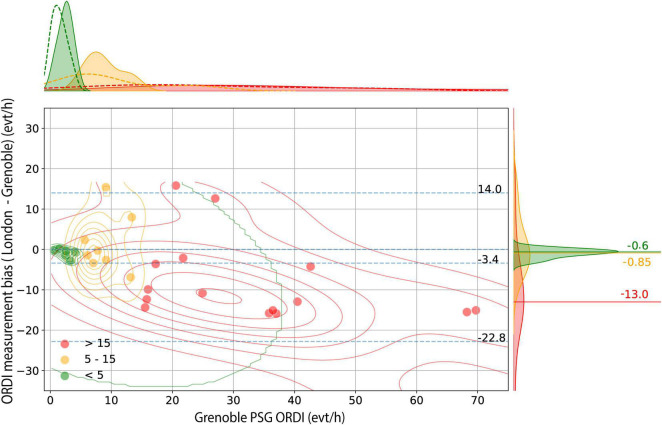
Bland-Altman analysis for London PSG-ORDI versus Grenoble PSG-ORDI. Bland-Altman plot shows the disagreement between average PSG-ORDI (London) and PSG-ORDI (Grenoble) (y axis) as a function of Grenoble PSG-ORDI (x axis), with individual cases stratified into three clinical groups. Bidimensional kernel density estimation plots are superimposed to show the joint distribution of measurement bias within each subgroup. The blue horizontal lines indicate the median, lower and upper bound (5th and 95th centiles) of the measurement bias in the whole sample. The distribution of the disagreement between the 2 methods, stratified by group, is shown on the right, with 3 horizontal lines indicating the median bias within each group. PSG: polysomnography; ORDI: obstructive respiratory disturbance index.

The PSG-ORDI from the two expert sleep centres were: London median 13.60 (IQR: 0.65 to 53.75) and Grenoble 15.9 (IQR: 2.15 to 69.00) events/hour. Overall, the London PSG-ORDI was lower: median −3.40 (95% CI −22.80 to + 14.00) events/hour compared to Grenoble (Figure 2). In patients with no OSA (ORDI < 5 events/hour) and those with mild OSA (ORDI 5–15 events/hour) there was a random and low median bias between the two centres of −0.60 (95% CI −2.58 to −0.03). However, in moderate-severe patients with ORDI > 15, the variation became more and unpredictable: mean bias −13.00, varying from −31.36 to + 13.22 events/hour.

There were significant linear correlations and high intraclass correlation coefficients among all the ORDI scores (*p*-values < 0.001) (see Appendix).

### Diagnostic Performance of the Mandibular Movement Monitoring System

**FIGURE 3:**
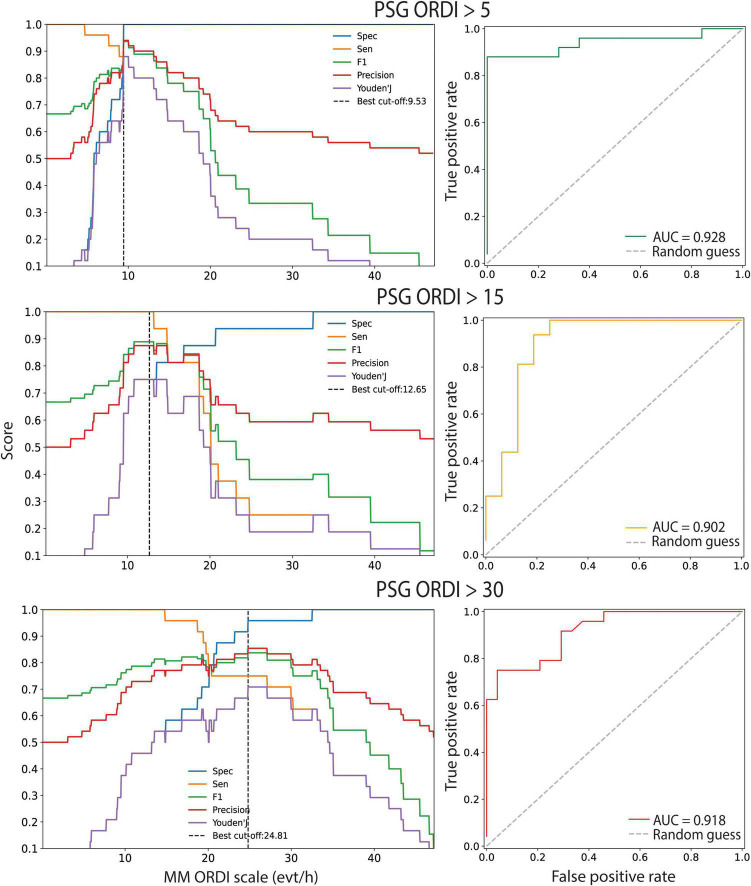
ROC curve analysis for MM-ORDI versus average PSG-ORDI. Cut-off calibration and ROC curves evaluating the global performance of 3 binary classification rules to detect patients with OSA with polysomnography-derived respiratory disturbance index (PSG-ORDI) of at least 5, 15 or 30 events/hour, using MM-ORDI. The 95% CIs of the area under the curve (AUC) were obtained by bootstrapping. The diagonal dotted line serves as a reference and shows the performance if OSA detection was made randomly. ROC: receiver operator characteristic; MM: mandibular movements; ORDI: obstructive respiratory disturbance index; OSA: obstructive sleep apnoea; 95% CIs: 95% confidence intervals; AUC: area under the curve.

The ROC analysis at OSA thresholds of 5, 15 and 30 events/hour corresponding to mild, moderate, and severe OSA is shown in Figure 3. The AUCs showed high global performance for each threshold; 0.928 (95% CI: 0.84 to 1.0), 0.902 (95% CI: 0.80 to 1.0) and 0.918 (95% CI: 0.79 to 1.0), respectively.

Optimal cut-offs were determined for the MM-ORDI. Mild OSA (PSG-ORDI > 5 events/hour) was detected with an optimal cut-off of 9.53 events/hour with a good balance between sensitivity and specificity (F1 = 0.94, BAC = 0.94). A previously reported cut-off of 7.63 events/hour yielded a high sensitivity, but lower specificity (Pépin et al., 2020). There was good diagnostic agreement for moderate OSA (>15 events/hour) using a cut-off of 12.65 events/hour (F1 = 0.89, BAC = 0.88). This cut-off was the same as observed previously (Pépin et al., 2020). MM-ORDI was also effective for detecting severe OSA (>30 events/hour) at cut-off of 24.81 events (F1 = 0.86, BAC = 0.87). At these cut-offs, the post-test probabilities of obtaining a true positive diagnosis were 100, 80, and 95% respectively (Table 1).

**TABLE 1:** Diagnostic performance of MM-ORDI versus PSG-ORDI.

	Detecting PSG-ORDI >5 events/hr	Detecting PSG-ORDI >15 events/hr	Detecting PSG-ORDI >30 events/hr
	
	Optimal cut-off (9.53)	Optimal cut-off (12.65)	Optimal cut-off (24.81)
Sensitivity	0.88	1.00	0.79
Specificity	1.00	0.75	0.96
F1	0.94	0.89	0.86
BAC	0.94	0.88	0.87
Positive predictive value	1.00	0.80	0.95
Negative predictive value	0.89	1.00	0.82
Positive likelihood ratio	Inf	4.00	19.0
Negative likelihood ratio	0.12	0.00	0.22
Youden J index	0.88	0.75	0.75

*BAC, balanced accuracy; MM, mandibular movement; ORDI, obstructive respiratory disturbance index; PSG, polysomnography*

## Discussion

Our study aimed to answer the question “is the Obstructive Respiratory Disturbance Index (ORDI) calculated using MM with machine learning analysis similar to the ORDI obtained using manually scored in-home PSG?” The main findings of this study are that the use of MM with machine learning analysis to diagnose OSA produced good agreement compared to in-home PSG-derived ORDI. The best agreement was observed at the mild end of the disease spectrum. Additionally, agreement in ORDI, between the MM monitor and in-home PSG, was similar to the agreement for the scoring of PSG between by the two expert centres.

Mandibular movement-derived respiratory disturbance measures and automated analysis demonstrated comparable performances than in-home PSG, suggesting that MM monitoring is an effective and practical way of testing for OSA in the patients’ own home. There is an expanding need for simple, automated tools for the diagnosis of OSA that can be used remotely (Randerath et al., 2018). However, it is important for these monitors to be accurate and reliable. Previously, the novel Sunrise device, using MM-derived respiratory disturbance measures and automated analysis, has been shown to have reliable agreement with PSG data recorded in-laboratory (Pépin et al., 2020). The findings of the present study show similar agreement using PSG recorded in the patients’ own home. Moreover, the high diagnostic performance, sensitivity and specificity compare favourably with other portable devices for the in-home detection of OSA (Mendonça et al., 2018). The advantages of using home-recorded data include reduced patient stresses, associated with travel and overnight hospital stays, plus a potential reduction in waiting times and clinical costs (Collop et al., 2007). A systematic review is currently underway to determine the cost-effectiveness of limited channel tests compared to laboratory and home PSG in diagnosing OSA (Natsky et al., 2021). Empirical studies support the use of limited channel tests carried out in the patients’ own home, suggesting similar efficacy, at lower costs, compared to PSG (Masa et al., 2014; Corral et al., 2017). These advantages of remote data collection, however, are balanced against the risk of technical failure. In the present study there were technical issues with the MM-monitor and smartphone application in 10% of studies, which is comparable to previously reported in-home PSG failure rate of 10–20% (Bruyneel and Ninane, 2014). This may be easily addressed by repeating limited channel studies at home over several nights, therefore reducing technical concerns and improving night-to-night variability estimation (Roeder et al., 2020, 2021).

In the current study, comparing PSG scored by experts from Grenoble and London, the ICC was 0.90. The magnitude of this difference was similar to the difference between the ORDI scored by the MM-analysis, compared to the mean ORDI calculated from both the Grenoble and London PSG scoring (ICC 0.85). The manual analysis of PSG data is time consuming. There is also variability between experts, despite the use of standardised scoring criteria. Inter-scorer agreement is generally between 70–80%, however, this figure increases when combined with automated scoring in an auto-edited approach (Magalang et al., 2013; Rosenberg and Van Hout, 2013; Younes et al., 2016). Machine learning for automatic sleep scoring presents many advantages including the removal of the subjectivity and unconscious bias associated with manual scoring. Machine learning algorithms have demonstrated a high level of accuracy and agreement, on average around 85%, between computer and manual scoring (Fiorillo et al., 2019).

Overall agreement between MM-ORDI and PSG-ORDI in the current study was good, with an overestimation of ORDI in mild disease. New technologies that do not use neurophysiological data to identify sleep, typically underestimate respiratory disturbance indices, such as AHI and ODI (Bianchi and Goparaju, 2017). This is due to the number of respiratory events being calculated across the total recording time, rather than total sleep time. Data from the ESADA study, showed that the AHI of patients investigated by polygraphy was approximately 30% lower, compared to patients investigated by PSG (Escourrou et al., 2015). Analysis of MM has previously been shown to reliably detect sleep and wake, which potentially leads to a more accurate calculation of ORDI (Senny et al., 2009, 2012; Maury et al., 2014).

In patients with severe OSA, however, the MM with machine learning analysis underestimated the ORDI. This is similar to results of a previous study, comparing MM analysis to in-lab PSG (Pépin et al., 2020). The scoring discrepancy in more severe OSA patients, may have been due to the use of the 2012 AASM recommended hypopnoea definition (Berry et al., 2012). Specifically, hypopnoeas can be scored when airflow reduction is followed either by a 3% oxygen desaturation, or an arousal from sleep. Therefore, cortical arousals detected by the occurrence of brisk and abrupt MM, typically associated with mouth closure, are reliably scored. However, hypopnoea events scored on PSG due to the presence of a 3% oxygen desaturation, may have been excluded by MM analysis. Ongoing algorithmic developments are likely, specifically to address the scoring of hypopnoeas. In a clinical setting, however, these patients represent the more severe end of the disease spectrum and therefore relatively small differences in ORDI may not impact diagnosis and treatment options, because treatment is usually recommended for patients with moderate-severe OSA (McDaid et al., 2009).

Recently, an international expert group have reinforced the need to move toward outcomes beyond the AHI for the diagnosis and classification of OSA (Randerath et al., 2018). Specifically, they recommend consideration of diagnostic criteria to reflect phenotypic variation. The use of a bio-signal such as MM may provide surrogate sleep data, alongside breathing data to improve the diagnosis of OSA.

### Strengths and Limitations

This is the first study to compare MM monitoring to PSG recording in the patients’ own home. Moreover, the OSA patients were recruited from a clinical referral population, enabling investigation of diagnosis across the disease spectrum. The PSG data was also independently analysed by experts in two centres. However, to fully interpret these data, several limitations need to be considered.

Firstly, the small sample size may have led to a type 2 error. Secondly, the increasing use of technology in healthcare can lead to issues associated with lack of access, either due to reduced internet availability in remote regions, or lack of familiarity e.g. in those who did not use mobile devices when they were younger, or other socioeconomic factors. There were three (7.5%) technical failures due to Bluetooth connection loss in the current study. Refinement of the technology and more access to training may ameliorate some of these issues. However, in-home studies require the ability to understand in-depth instructions, thus information must be given to patients in a clear, concise format (Medicines and Healthcare products Regulatory Agency [MHRA], 2021).

## Conclusion and Implications

For future routine clinical practice, MM with machine learning analysis had good agreement with manually scored PSG recorded in the patients’ own home and is a promising option for home-based, automated assessment for OSA. Further studies will evaluate the use of the monitor in different care pathways, the patient experience and cost-effectiveness of this new technology. For policy makers, it is time to consider reimbursement and large-scale development of such simplified techniques for sleep apnoea diagnosis.

## Data Availability Statement

The raw data supporting the conclusions of this article will be made available by the authors, without undue reservation.

## Ethics Statement

The studies involving human participants were reviewed and approved by Comité de Protection des Personnes, Sud-Ouest et Outremer III, Bordeaux, France. The patients/participants provided their written informed consent to participate in this study.

## Author Contributions

JLP, JBM, and MM contributed to the conception and the design of the study and had full access to all of the data in the study and take responsibility for the integrity of the data and the accuracy of the data analysis. RBM and RTe out carried the clinical research and organised the database. NL-D performed the statistical analysis. JK wrote the first draft of the manuscript. RBM and MJF wrote sections of the manuscript. JLP, JBM, MM, MJF, and RTa: critically revised the manuscript for important intellectual content. All authors contributed to manuscript revision, read and approved the submitted version.

## Conflict of Interest

The authors declare that the research was conducted in the absence of any commercial or financial relationships that could be construed as a potential conflict of interest.

## Publisher’s Note

All claims expressed in this article are solely those of the authors and do not necessarily represent those of their affiliated organizations, or those of the publisher, the editors and the reviewers. Any product that may be evaluated in this article, or claim that may be made by its manufacturer, is not guaranteed or endorsed by the publisher.

## Appendix

### Machine Learning Analysis

The obstructive respiratory disturbance index (ORDI) was calculated based on a combination of awake/sleep and respiratory effort/arousal/quiet sleep classification tasks. These are handled by stacked classifiers: a binary Random Forest classifier for awake/sleep detection based and a multiclass Random Forest classifier for respiratory effort/arousal/quiet sleep detection based on 30 s epochs.

Input features consisted of a combination of axes of the accelerometer/gyroscope, processing modes (filter with several frequency bands, moving average) and statistical functions. The statistics applied to the above features were tendency toward centrality (mean, median), extreme values (min, max), quartiles, standard deviation, as well the normal standardised version of all above features.

The hyper-parameters of Random Forest classifiers were optimised through a grid-search based 10 × 10 cross-validation, which aimed to minimise the logarithmic losses. Following hyper-parameters were considered for tuning: tree depth, minimum sample split and tree number.

Choice of the machine learning (ML) approaches:

Machine learning approaches can be classified into 2 main categories: (a) deep learning or (b) conventional methods on structured data, depending on two factors: (1) how to extract input features from the raw signals? and (2) what algorithm should be used?

Based on literature review and previous experiments, there remains uncertainty about the superiority of any specific algorithm among tree-based ensemble algorithms (XGBoost, Random Forest) or deep learning models.

The only advantage of deep learning framework is allowing for automatic feature extraction from raw signal. However, on a well determined problem, with appropriate pre-processing techniques and carefully validated labels, there would be no difference in performance between deep learning and tree-based models.

Instead of using the deep learning models, it was decided to use the features extraction framework, which allows better control and understanding of input data compared to black-box models like convolutional neural networks in deep learning.

Due to the large training data size, complexity of the output and high dimensionality of input features, Random Forest algorithm has been adopted. This algorithm offers several advantages over the classical methods (linear discriminant, support vector machine), including better performance, high efficiency in computation, ability of detecting important features, fast training, and execution speeds. Compared with XGboost, the Random Forest model would have the same level of performance on tabular data, with less complexity in tuning process, as they have less hyper-parameter than XGBoost.
