# Supplementary material for: Diagnosis of Sleep Apnoea Using a Mandibular Monitor and Machine Learning Analysis: One-Night Agreement Compared to in-Home Polysomnography
Source: Front Neurosci. 2022 Mar 15;16:726880. doi: 10.3389/fnins.2022.726880 (PMC8965001; doi:10.3389/fnins.2022.726880)
Supplement: Supplementary file 1 [file Data_Sheet_1.docx]

**SUPPLEMENT**

There was good agreement between the two sleep laboratories in Grenoble and London was noticed for PSG scoring. However, an underestimation by MM analysis was detected for PSG-ORDI>50 events/hour. (Figure 1)

Results of ICC analysis indicate a high covariance between MM-ORDI and PSG-ORDI (calculated as the mean results of two scorers in London and Grenoble). (Figure 2)

There was strong and significative linear correlation among ORDI scores by 3 different methods (Table S2 and Figure S2).

**Figure Legends**

**Figure S1: Distribution of ORDI scores**

Kernel density plots showing the distribution of ORDI scores.

ORDI: obstructive respiratory disturbance index; MM: mandibular movements; PSG: polysomnography

**Figure S2: Linear regression plots visualising the relationship between the ORDI scores**

For each panel, x axis indicates the predictor and y axis indicates the predicated value. Each point represents a single case. The dotted traces represent the identity line or a perfect equivalence between 2 variables.

ORDI: obstructive respiratory disturbance index; MM: mandibular movements; PSG: polysomnography


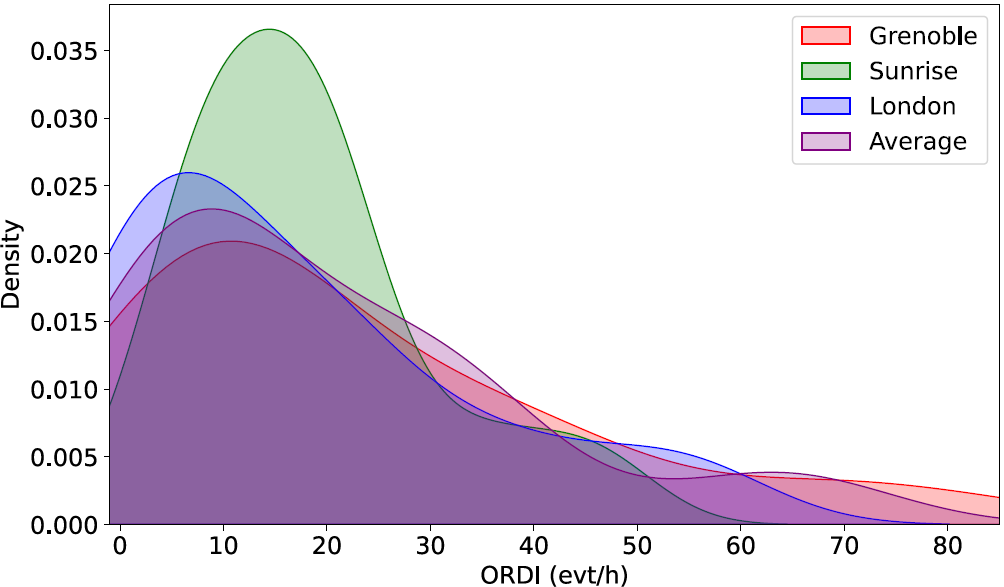


**Figure S1: Distribution of ORDI scores**


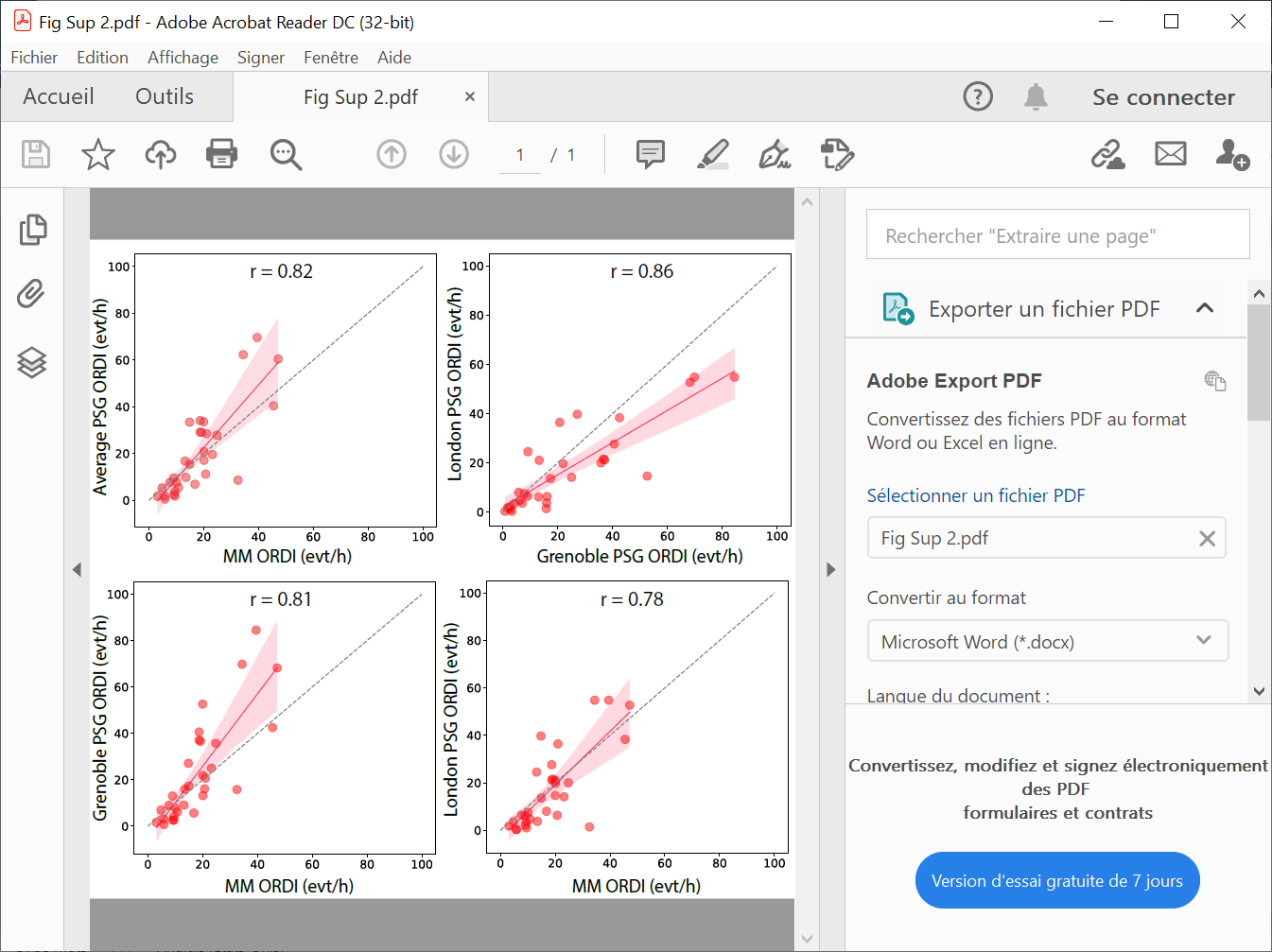


**Figure S2: Linear regression plots visualising the relationship between the ORDI scores**

**Tables**

**Table S1: Distribution of ORDI scores.**

| Indices | n | Mean | Min | 5% | Median | 95% | Max |
| --- | --- | --- | --- | --- | --- | --- | --- |
| Grenoble PSG-ORDI | 31 | 23.01 | 0.70 | 2.15 | 15.90 | 73.48 | 84.50 |
| London PSG-ORDI | 31 | 17.13 | 0.30 | 0.65 | 13.60 | 54.80 | 54.80 |
| Average PSG-ORDI | 31 | 20.07 | 0.50 | 1.75 | 15.45 | 64.14 | 69.65 |
| MM ORDI | 31 | 18.20 | 3.10 | 5.30 | 16.80 | 45.93 | 47.20 |

MM: mandibular movement; ORDI: obstructive respiratory disturbance index; PSG: polysomnography

**Table S2: Correlation matrix of ORDI scores**

|  | Grenoble PSG-ORDI | London PSG-ORDI | Average PSG-ORDI | MM-ORDI |
| --- | --- | --- | --- | --- |
| Grenoble PSG-ORDI | _ | 0.85 | 0.97 | 0.81 |
| London PSG-ORDI | 0.85 | _ | 0.95 | 0.77 |
| Average PSG-ORDI | 0.97 | 0.95 | _ | 0.82 |
| MM-ORDI | 0.81 | 0.77 | 0.82 | _ |

All comparisons are significative with p values < 0.001

ORDI: obstructive respiratory disturbance index; MM: mandibular movements; PSG: polysomnography

**Table S3: Intraclass correlation coefficients analysis**

| ICC(3,2) | Estimated | 5th | 95th | p value |
| --- | --- | --- | --- | --- |
| Mean PSG-ORDI vs MM-ORDI | 0.85 | 0.56 | 0.85 | <0.001 |
| Grenoble PSG-ORDI vs MM-ORDI | 0.80 | 0.63 | 0.89 | <0.001 |
| Grenoble PSG-ORDI vs London PSG-ORDI | 0.90 | 0.82 | 0.95 | <0.001 |
| London PSG-ORDI vs MM-ORDI | 0.84 | 0.70 | 0.91 | <0.001 |

ICC (3,2) corresponds to a two-way mixed, average score ICC, meaning that 2 fixed scorers are defined. Each subject is measured by both scorers, the reliability is applied to a context where measures of 2 scorers will be averaged for each subject.

ORDI: obstructive respiratory disturbance index; MM: mandibular movements; PSG: polysomnography; ICC: intraclass correlation coefficient
